# Prognostic Impact of Radiological Consolidation Tumor Ratio in Clinical Stage IA Pulmonary Ground Glass Opacities

**DOI:** 10.3389/fonc.2021.616149

**Published:** 2021-04-12

**Authors:** Junjie Xi, Jiacheng Yin, Jiaqi Liang, Cheng Zhan, Wei Jiang, Zongwu Lin, Songtao Xu, Qun Wang

**Affiliations:** Department of Thoracic Surgery, Zhongshan Hospital, Fudan University, Shanghai, China

**Keywords:** lung cancer, early-stage, computed tomography, ground-glass opacities, consolidation

## Abstract

**Objectives:**

Our study aimed to validate pathologic findings of ground-glass nodules (GGOs) of different consolidation tumor ratios (CTRs), and to explore whether GGOs could be stratified according to CTR with an increment of 0.25 based on its prognostic role.

**Methods:**

We retrospectively evaluated patients with clinical stage IA GGOs who underwent curative resection between 2011 and 2016. The patients were divided into 4 groups according to CTR step by 0.25. Cumulative survival rates were calculated by the Kaplan-Meier method. Univariate and multivariate Cox regression analyses were conducted to obtain the risk factors on relapse-free survival (RFS). The surv_function of the R package survminer was used to determine the optimal cutoff value. Receiver operating characteristic (ROC) analysis was generated to validate optimal cutoff points of factors.

**Results:**

A total of 862 patients (608 women; median age, 59y) were included, with 442 patients in group A (CTR ≤ 0.25), 210 patients in group B (0.25<CTR ≤ 0.5), 173 patients in group C (0.5<CTR ≤ 0.75), and 37 patients in group D (0.75<CTR<1). The rate of adenocarcinoma *in situ* (AIS) or minimally invasive adenocarcinoma (MIA) in group A (70.6%) was much higher than other three groups (p<0.001). Multivariable Cox regression revealed that CTR (HR, 1.865; 95%CI, 1.312-2.650; *p* = 0.001) and lymph node metastasis (HR, 10.407; 95%CI, 1.957-55.343; *p* = 0.006) were independent prognostic factors for recurrence free survival. In addition, CTR was the only risk factor for the presence of micropapillary or solid pattern (OR=133.9, 95%CI:32.2-556.2, *P*<0.001) and lymph node metastasis (OR=292498.8, 95%CI:1.2-7.4×10^10^, *P*=0.047). Paired comparison showed that rate of presence of micropapillary or solid pattern was highest in group D, followed by group C and group A/B (p<0.001). Lymph node metastasis occurred in group D only (*p*=0.002).

**Conclusions:**

CTR is an independent prognostic factor for clinical stage IA lung adenocarcinoma manifesting as GGO in CT scan. Radiologic cutoffs of CTR 0.50 and 0.75 were able to subdivide patients with different prognosis.

## Introduction

Ground glass opacity (GGO) is a radiological finding in computed tomography (CT) with a hazy opacity that does not obscure the underlying bronchial structures or pulmonary vessels (1–3). Lung adenocarcinoma with GGO component is correlated with excellent prognosis (4). Both consolidation size and consolidation tumor ratio (CTR) were reported to be prognostic factors for GGOs (5–7).

Previous studies revealed that GGO dominant (CTR ≤ 0.5) part-solid nodules were less invasive than solid dominant (CTR>0.5) part-solid nodules (8–14). In the 2015 World Health Organization classification of lung tumors (15), micropapillary and solid components in adenocarcinoma represent poor differentiation and worse biology behavior. It has been reported that these two poor differentiated components correlate with poor prognosis (16–19), and it has been verified in clinical stage I non-small-cell lung cancer as well (20–22). Pathologic components of lung adenocarcinoma might transform into prognostic information in the long term to some extent. Few studies have investigated the pathologic subtypes of GGOs of different CTRs, with an increment of 0.25. Our study is to investigate prognostic factors of GGOs, and then to explore whether GGOs should be studied according to CTR with an increment of 0.25, considering both survival and pathology.

## Patients and Methods

This study was conducted in accordance with the amended Declaration of Helsinki. Ethics committee on human research of Zhongshan Hospital approved the protocol (approval number: B2019-232R), and written informed consent was obtained from all patients before surgery for the use of surgical samples and clinical information for medical research.

### Patient Selection

We retrospectively reviewed the records of patients with GGOs who underwent curative resection at our institute between January 2011 and December 2016. All patients received thin-section CT scan (collimation ≤1.5mm) before surgery. For patients with solid component ≥6mm in the lung window, PET/CT was regularly recommended. Most patients underwent standard lobectomy, while sublobar resection (segmentectomy and wedge resection) was performed for a section of patients with tumors ≤ 2cm. A minimum of three N2 stations sampled or complete lymph node dissection was a routine schedule for all patients. Inclusion criteria were as follows: (1) GGO with maximum consolidation diameter ≤ 3cm in the lung window, (2) clinically no lymph node metastasis (shortest diameter of hilar or mediastinal lymph nodes less than 1.0cm on CT scan or no positive fluorodeoxyglucose uptake of hilar or mediastinal lymph nodes on PET/CT), (3) pathologically confirmed primary lung adenocarcinoma, and (4) R0 (complete) resection. Cases with no pathologic subtype data were excluded. Finally, 862 stage IA patients were included.

The patients were divided into 4 groups according to CTR: group A (CTR ≤ 0.25), group B (0.25<CTR ≤ 0.5), group C (0.5<CTR ≤ 0.75), and group D (0.75<CTR<1).

### CT Measurement

**Figure 1:**
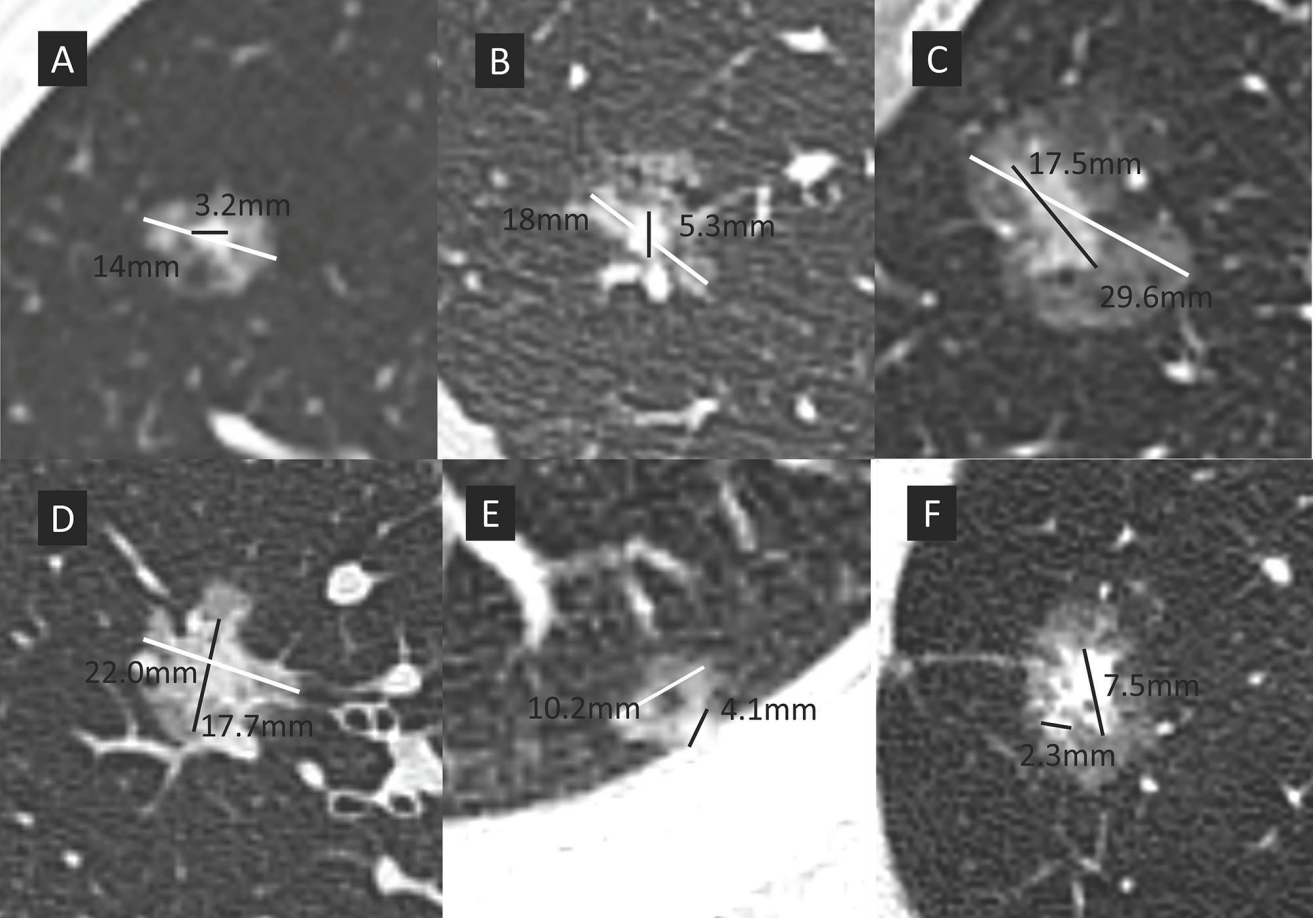
The maximum size of consolidation is divided by the maximum tumor size in the lung window to give the consolidation tumor ratio (CTR). White line represents the maximum overall nodule dimension, black line represents the long axis of the solid component. Patients were divided into 4 groups according to CTR. **(A)** group A, CTR ≤ 0.25; **(B)** group B, 0.25<CTR ≤ 0.5; **(C)** group C, 0.5<CTR ≤ 0.75; **(D)** group D, 0.75<CTR<1; **(E)** a sub-solid nodule with cystic component, the solid components abuts the chest wall and cystic components, makes the accurate measurement challenging. The largest solid dimension was selected for measurement; **(F)** two separate solid components existed in the same nodule (maximum diameter, 7.5mm). Only the largest component needs to be measured in nodules with multiple solid components.

The lung windows were set at a window width of 1500 Hounsfield units (HU) and a window level -500 HU. GGO is defined as a hazy opacity in lung without obscuring the underlying bronchial structures or pulmonary vessels. Pathologically, GGO mainly turns out to be lepidic but also non-lepidic growth patterns in lung adenocarcinomas. The consolidation component is defined as an area of increased opacity that completely obscures the underlying bronchial structures and pulmonary vessels. The longest diameters of the solid portion and total tumor size in the lung window were measured, respectively. The CTR was defined as the ratio of the maximum size of consolidation to the maximum tumor size in the lung window (**Figure 1**). For multiple GGOs, the dominant lesions were investigated. In circumstances that multiple solid components existed in one pulmonary nodule, the largest consolidation was measured. Two independent radiologists with at least 5-year experience reviewed the CT scans and determined tumor sizes. 102 of 862 nodules (11.8% disagreement) were discordant in the solid component size. The nodules with discrepancy were adjudicated by the third radiologist (with 15-year experience in chest radiology) and final results were settled by consensus.

### Pathologic Examination

All resection specimens were formalin-fixed and stained with hematoxylin and eosin. Pleural invasion was established using elastin stains in case that it was difficult to diagnose pleural invasion. Pathologic diagnosis was made according to the 2011 International Association for the Study of Lung Cancer/American Thoracic Society/European Respiratory Society (IASCL/ATS/ERS) classification. Each histological component (lepidic, acinar, papillary, micropapillary, and solid) was recorded. The predominant pattern was defined as the pattern with the largest percentage.

### Follow-Up Protocol

The initial postoperative surveillance schedule includes a chest CT scan and a history and physical examination (H&P) examination every 3-6 months for first 2 years, followed by an annual chest CT and an H&P for subsequent years. Brain contrast-enhanced magnetic resonance imaging (MRI) and emission computed tomography (ECT) bone scan were performed every 6 months and 12 months respectively for all patients in the first 3 years and upon occurrence of the corresponding symptoms.

### Statistical Analysis

Differences in categorical variables were compared using chi-square test or Fisher’s exact test. Continuous variables were compared using paired t test. Bonferroni adjustments were included for multiple comparisons. Estimation of survival curves of recurrence free survival (RFS) was generated by the Kaplan-Meier method, and survival curves were compared using the log-rank test. Logistic regression was used for dichotomous outcomes. Univariate and multivariate analyses using Cox’s proportional hazard model were conducted to obtain the risk factors for relapse-free survival (RFS). Factors with P- value <0.10 were included in multivariate analysis. The surv_cutpoint function in R package “survminer” was applied to determine the optimal cutoff of CTR for RFS. ROC analyses were generated to validate the cutoff value of CTR for RFS and calculate the optimal cutoff values of CTR for micropapillary/solid pathologic subtypes and lymph node metastasis. All statistical tests were 2-sided and statistical significance was defined as P < 0.05. Statistical analysis was conducted using Statistical Package for Social Sciences (SPSS) 20.0 software (SPSS Inc., Chicago, IL, USA), GraphPrism 5.0 software (GraphPad Software, Inc., La Jolla, CA, USA) and R version 4.0.3 (http://www.r-project.org/). The R package included survival, survminer and ggplot2.

## Results

### Clinicopathological Characteristics

The clinicopathological characteristics of the patients are demonstrated in **Table 1**. The majority of the patients were female, with no significant difference achieved among four groups (P=0.06). With higher CTR, lobectomy was chosen more frequently than sublobar resection (P<0.001). Both whole tumor size and consolidation size were higher in group C and group D. There was no significant difference in the rate of smoking history between the four groups (P=0.333). As to pathologic subtype, the rate of adenocarcinoma *in situ* (AIS) or minimally invasive adenocarcinoma (MIA) was 70.6% in group A, which was much higher than the other three groups (P<0.001).

**Table 1:** Clinicopathological Characteristics of Patients.

	Group A	Group B	Group C	Group D	Overall	*P Value*
**Gender**						0.060
female	328 (74.2%)	135 (64.3%)	121 (69.9%)	24 (64.9%)	608(70.5%)	
male	114 (25.8%)	75 (35.7%)	52 (30.1%)	13 (35.1%)	254 (29.5%)	
**Age**						<0.001
Mean (SD)	53.7 (11.5)	58.6 (10.6)	60.3 (9.4)	61.3 (9.8)	56.6 (11.1)	
Median	56.0	59.5	61.0	60.0	59.0	
**Surgical mode**						<0.001
lobectomy	163 (36.9%)	116 (55.2%)	115 (66.7%)	26 (70.3%)	420 (48.7%)	
sublobar resection	279(63.1%)	94 (44.8%)	58 (33.3%)	11 (29.7%)	442 (51.3%)	
**Surgical approach**						0.005
VATS	440 (99.5%)	208 (99.0%)	167 (96.5%)	35 (94.6%)	850 (98.6%)	
thoracotomy	2 (0.5%)	2 (1.0%)	6 (3.5%)	2 (5.4%)	12 (1.4%)	
**Whole tumor size**						<0.001
Mean (SD)	10.5 (4.6)	15.9 (6.3)	18.7 (7.5)	18.2 (6.9)	13.7 (6.8)	
Median	9.0	15.0	17.0	17.0	12.0	
**Consolidation size**						<0.001
Mean (SD)	0.4 (1.1)	6.3 (2.8)	11.5 (4.8)	14.9 (5.5)	4.7 (5.7)	
Median	0.0	6.0	10.0	13.0	3.0	
**CTR**						<0.001
Mean (SD)	0.03 (0.07)	0.39 (0.07)	0.62 (0.07)	0.83 (0.05)	0.27 (0.28)	
Median	0.00	0.39	0.60	0.82	0.25	
**Smoking history**						0.333
no smoking history	413 (93.4%)	194 (92.4%)	154 (89.0%)	34 (91.9%)	795 (92.2%)	
current smoker or have smoking history	29 (6.6%)	16 (7.6%)	19 (11.0%)	3 (8.1%)	67 (7.8%)	
**Pathologic subtype**						<0.001
AIS	101 (22.9%)	7 (3.3%)	2 (1.2%)	0 (0%)	110 (12.8%)	
MIA	221 (47.7%)	36 (17.1%)	19 (11.0%)	2 (5.4%)	268 (31.1%)	
Lepidic dominant	28 (6.3%)	27 (12.9%)	22 (12.7%)	2 (5.4%)	79 (9.2%)	
Acinar dominant	99 (22.4%)	135 (64.3%)	125 (72.3%)	29 (78.4%)	388 (45.0%)	
Papillary dominant	3 (0.7%)	5 (2.4%)	5 (2.9%)	4 (10.8%)	17 (2.0%)	
**Differentiation**						<0.001
well/moderate	436 (98.6%)	202 (96.2%)	156 (90.2%)	23 (62.2%)	817 (94.8%)	
poorly	6 (1.4%)	8 (3.8%)	17 (9.8%)	14 (37.8%)	45 (5.2%)	
**Pleural invasion**						<0.001
no pleural invasion	638 (99.1%)	189 (90.0%)	142 (82.1%)	27 (73.0%)	796 (92.3%)	
pleural invasion	4 (0.9%)	21 (10.0%)	31 (17.9%)	10 (27.0%)	66 (7.7%)	
**Lymph node metastasis**						<0.001
negative	442 (100%)	210 (100%)	173 (100%)	35 (94.6%)	860 (99.8%)	
positive	0 (0%)	0 (0%)	0 (0%)	2 (5.4%)	2 (0.2%)	

SD, stand deviation; VATS, video-assisted thoracic surgery; CTR, consolidation/tumor ratio; AIS, adenocarcinoma in situ; MIA, minimally invasive adenocarcinoma.

### Cox Regression

Univariate and multivariate analyses results were summarized in **Table 2**. Age, gender, surgical mode, surgical approach, whole tumor size, consolidation size, CTR, smoking history, differentiation, pleural invasion, and lymph node metastasis were included in the analysis. For RFS, surgical approach, whole tumor size, consolidation size, CTR, differentiation, pleural invasion, and lymph node metastasis were included in the multivariate analysis. CTR (HR, 1.865; 95%CI, 1.312-2.650; *p* = 0.001) and lymph node metastasis (HR, 10.407; 95%CI, 1.957-55.343; *p* = 0.006) were identified as independent prognostic factors.

**Table 2:** Univariate and Multivariate Survival Analysis for Relapse-free Survival.

Variables	Univariate			Multivariate		
	HR	95%CI	*P* Value	HR	95%CI	*P* Value
Age	1.051	0.987-1.119	0.118			
Gender						
female	1					
male	0.881	0.234-3.322	0.851			
Surgical mode						
lobectomy	1					
sublobar resection	0.595	0.173-2.042	0.409			
Surgical approach						
VATS	1					
thoracotomy	5.839	0.733-46.530	0.096			
Whole tumor size	1.064	0.997-1.136	0.061			
Consolidation size	1.147	1.073-1.227	<0.001			
CTR	2.011	1.420-2.848	<0.001	1.865	1.312-2.650	0.001
Smoking history						
no smoking history	1					
current smoker or have smoking history	0.044	0.000-915.826	0.539			
Differentiation						
AIS/MIA/IAD without micropapillary or solid component	1					
IAD with micropapillary and/or solid component	8.326	2.172-31.916	0.002			
Pleural invasion						
no pleural invasion	1					
pleural invasion	9.287	2.823-30.551	<0.001			
Lymph node metastasis						
negative	1			1		
positive	75.776	15.608-367.889	<0.001	10.407	1.957-55.343	0.006

HR, hazard ratio; CI, confidence interval; VATS, video-assisted thoracic surgery; CTR, consolidation/tumor ratio; Well/moderate differentiation.

AIS, adenocarcinoma in situ; MIA, minimally invasive adenocarcinoma; IAD, invasive adenocarcinoma.

### Survival Analysis

**Figure 2:**
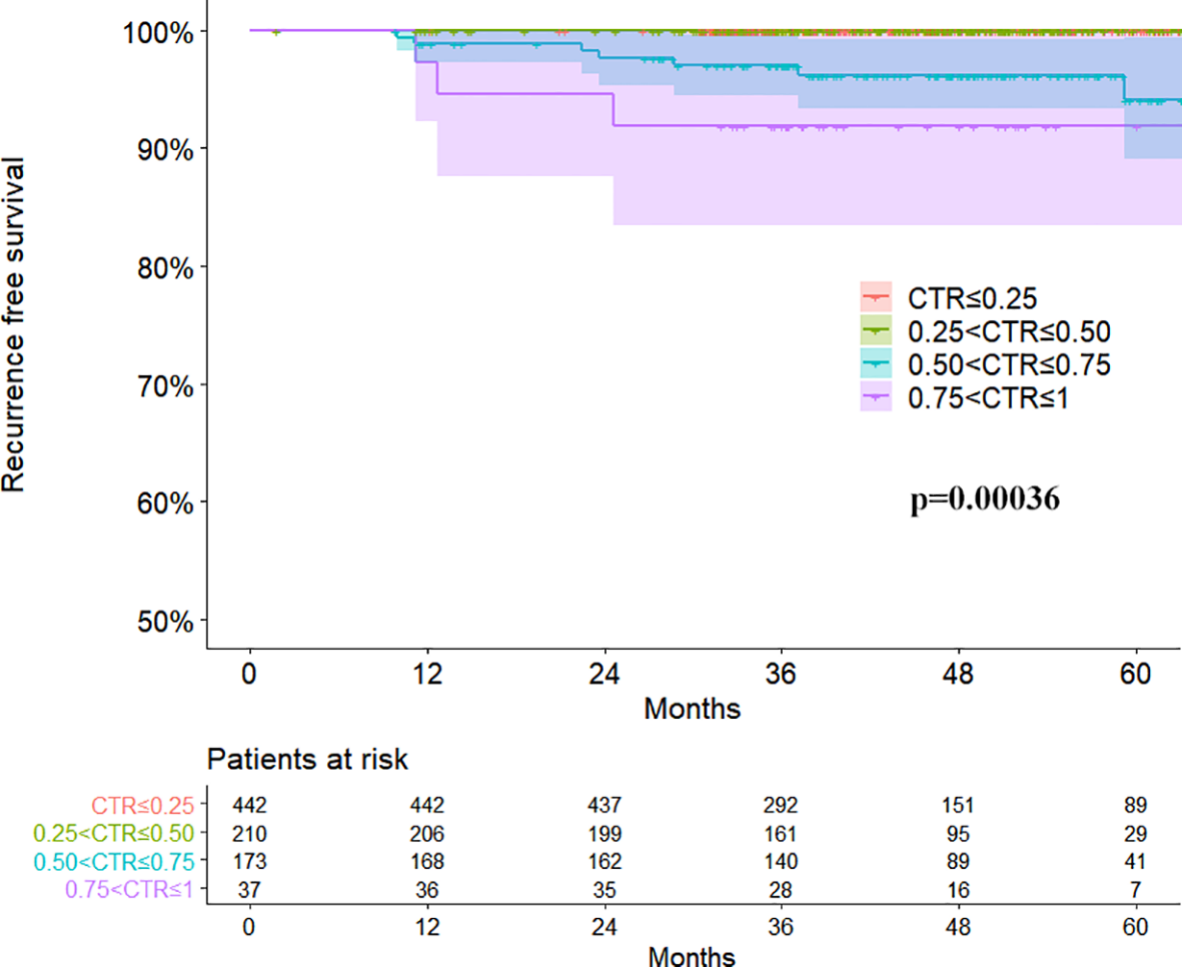
Recurrence free survival curves of four groups divided by consolidation tumor ratio in clinical stage IA lung adenocarcinomas.

**Figure 3:**
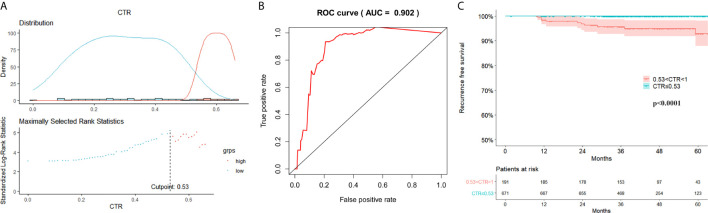
Optimal cutoff value of consolidation tumor ratio for recurrence free survival (RFS). **(A)** Surv_function provided a cutoff value of consolidation tumor ratio (CTR) 0.53 that corresponded to the most significant relation with RFS; **(B)** In receiver operating characteristics analysis, the optimum cutoff value of CTR for RFS was 0.53, area under the ROC curve (AUC) was 0.902; **(C)** The 5-year RFS was significantly different between groups CTR ≤ 0.53 and 0.53<CTR<1 (P < 0.001).

The follow-up duration ranged from 2 to 108 months (mean: 47 months). The RFS survival curves of 4 groups were demonstrated in **Figure 2**. There was no relapse in group A and group B. The log-rank test between group A/B and group C/D revealed a significant difference in RFS (p<0.001). The difference turned out to be insignificant between group C and group D (P=0.096). Surv_function was used to determine the optimal cutoff value of CTR for RFS, which is 0.53 (**Figure 3A**). ROC analysis also indicated that the optimal cutoff point of CTR for RFS was 0.53, with the area under the ROC curve (AUC) of 0.902 (**Figure 3B**). There was significant difference in 5-year RFS rate between groups CTR ≤ 0.53 and 0.53<CTR<1 (P<0.0001) (**Figure 3C**).

### Logistic Regression Analysis

To investigate risk factors for the presence of micropapillary or solid pattern and lymph node metastasis, logistic regression analyses were performed. Preoperative parameters were included, such as age, gender, whole tumor size, consolidation size, CTR, and smoking history. CTR was the only risk factor for the presence of micropapillary or solid pattern (OR=133.9, 95%CI:32.2-556.2, *P*<0.001) and lymph node metastasis (OR=292498.8, 95%CI:1.2-7.4×10^10^, *P*=0.047).

### Differences in Presence of Micropapillary/Solid Component and Lymph Node Metastasis

**Figure 4:**
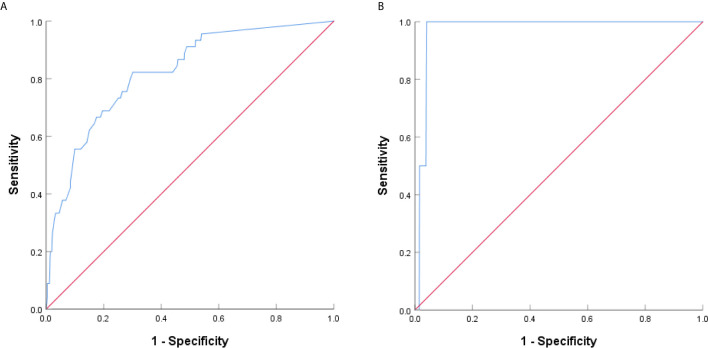
ROC curve analysis based on CTR for poor differentiation and lymph node metastasis. **(A)** The AUC indicated the diagnostic power of CTR for poor differentiation, AUC was 0.822 (95% confidence interval (CI): 0.760-0.883), with a sensitivity of 82.2% and specificity of 69.9% by the Youden’s index. **(B)** The AUC indicated the diagnostic power of CTR for lymph node metastasis, AUC was 0.972 (95% confidence interval (CI): 0.953-0.992), with a sensitivity of 100.0% and specificity of 95.9% by the Youden’s index.

The presence of micropapillary/solid component of the 4 groups was showed in **Table 3**. The difference between group A/B and group C/D was significant (*P*<0.001). Paired comparison showed that the presence rate of micropapillary/solid component in group D was significantly higher than other 3 groups (P<0.001), the presence rate of micropapillary/solid component in group C was significantly higher than group B (P=0.030) and group A (P<0.001), while there was no significant difference between group A and group B (P=0.084). ROC curve revealed the optimal cutoff value of CTR for poor differentiation was 0.47 (**Figure 4A**).

**Table 3:** Presence of micropapillary/solid component in 4 groups.

		Group			
		A	B	C	D
Micropapillary/Solid component	not present	436(98.6%)	202(96.2%)	156(90.2%)	23(62.2%)
	present	6(1.4%)	8(3.8%)	17(9.8%)	14(37.8%)

Lymph node metastasis occurred in group D only (**Table 4**), and the difference was significant (*P*=0.002). Paired comparison showed that lymph node metastasis rate in group D was significantly higher than the other 3 groups, while there was no significant difference between group A, group B, and group C. ROC curve revealed the optimal cutoff value of CTR for lymph node metastasis was 0.76 (**Figure 4B**).

**Table 4:** Lymph node metastasis in 4 groups.

		Group			
		A	B	C	D
Lymph node metastasis	No	442(100.0%)	210(100.0%)	173(100.0%)	35 (94.6%)
	Yes	0(0.0%)	0(0.0%)	0(0.0%)	2(5.4%)

### Comment

The incidence of GGOs has been rising in recent years with the widespread use of CT scan, especially thin-section CT scan. A majority of resected GGOs were confirmed to be early-stage lung adenocarcinoma or atypical adenomatous hyperplasia. In both retrospective and prospective studies, patients with GGO lesions have a better survival rate than patients with pure solid lung cancer after surgical resection (2, 3, 23–26). For pure GGO lesion patients, the lung cancer specific survival rate was reported to be 100% (24, 25, 27).

Even though the prognosis for GGO is good, there remains recurrence and lung cancer specific death. It is important to know the prognostic factors for GGOs, which may help determine the treatment regimen and resection extension. CTR has been considered to be associated with outcomes in pulmonary GGOs. In some retrospective studies, CTR of 0.5 is suggested as a cutoff value for pathological noninvasiveness in GGO lesions (8–13). Japan Clinical Oncology Group (JCOG) 0201 (28), a prospective radiological study, suggested that noninvasive adenocarcinoma could be defined as an adenocarcinoma ≤2cm with CTR ≤ 0.25. The survival outcomes of JCOG 0201 revealed that the criteria of nodules ≤3cm with CTR ≤0.5 also identify a group of patients with excellent prognosis (29). Thus, the eligibility criteria for JCOG 0802, a prospective clinical trial to compare lobectomy and sublobar resection, were changed to be tumor ≤2cm with CTR>0.5, instead of CTR>0.25 (30). In some retrospective studies, consolidation tumor size was recognized as a prognostic factor as well (5, 31). In the eighth TNM staging system (7), the clinical T stage of part-solid GGO is suggested to be determined by the solid component. Whereas, Hattori and his colleagues (14) found that neither consolidation tumor size nor CTR was associated with overall survival in part-solid lung cancer. In our study, CTR was found to be an independent prognostic factor for RFS in the multivariate COX regression analysis.

As CTR is an independent prognostic factor for GGO, it is then reasonable to study how to divide GGOs with CTR. CTR cutoff value of 0.5 is commonly used to divide GGOs into GGO-predominant nodules and solid-predominant nodules. In our study, the RFS survival rate of GGO-predominant nodules (group A and B, no relapse) was significantly higher than that of solid-predominant nodules (group C and D). The survival of group D was worse than group C, but the difference was not significant (P=0.096). Hattori and his colleagues (26) retrospectively analyzed 497 clinical stage IA radiologic invasive adenocarcinomas, in which 177 nodules were solid-predominant part-solid GGOs. When the solid-predominant part-solid GGOs were divided into two groups with 0.75 of CTR as cutoff, the 5-year overall survival was equivalent in the two groups (95.3% versus 96.8%, *p* = 0.703). These results may indicate that 0.5 is a good cutoff for CTR, and neither GGO-predominant GGOs nor solid-predominant GGOs should be divided further. However, as tumors with GGO component have excellent prognosis, studies for GGO lesions demand longer follow-up duration and larger sample size compared with studies for solid tumors, if overall survival or relapse-free survival is chosen to be the exclusive endpoints. Besides, as average life expectancy has been increased in China for years, patients cannot accept a similar 5-year survival rate but a worse 10-year survival rate.

In 1995, Noguchi and his colleagues reviewed 236 surgically resected small peripheral adenocarcinomas ≤2 cm and proposed a pathologic classification of 6 types based on tumor growth patterns (32). In Noguchi’s classification, type D is poorly differentiated adenocarcinoma with lower survival rate than type A, B or C. In 2011, IASLC/ATS/ERS proposed a new histological classification of pulmonary adenocarcinoma (19). Micropapillary subtype was introduced to the new classification since micropapillary component was a poor prognostic factor (20, 33). Even in early-stage lung cancer, the presence of micropapillary and solid pattern still correlated with poor survival in several studies (21, 22, 34, 35). The poorly-differentiated patterns represent worse biological behavior of the tumors to some extent. Unlike survival data, pathological information can be collected in short term without concerning insufficient follow-up duration.

To determine whether CTR is associated with the presence of micropapillary or solid pattern and lymph node metastasis, we performed logistic regression analyses, and confirmed that CTR was the only risk factor for the presence of micropapillary or solid pattern (OR=133.9, 95%CI:32.2-556.2, *P*<0.001) and lymph node metastasis (OR=292498.8, 95%CI:1.2-7.4×10^10^, *P*=0.047). Further analysis showed that the rate of presence of micropapillary or solid pattern was highest in group D, followed by group C and group A/B; the rate of lymph node metastasis was significantly higher in group D than other 3 groups. The biological behavior was different between group A/B, group C and group D. To better study solid-predominant GGOs, the subdivision seems necessary. Considering the good prognosis, sublobar resection could be enough for GGOs. Recent studies have reported similar survival outcomes of part-solid adenocarcinoma treated with sublobar resection and lobectomy (2, 26). However, heterogeneity exists among solid dominant GGOs. As a visual and accessible variable in the clinical work, CTR might provide prognostic implications for appropriate candidates of sublobar resection. Prospective clinical trials are warranted to validate this.

There are several limitations to this study. Firstly, this is a retrospective study that may inevitably lead to bias. Secondly, the sample size for group D is small. Thirdly, longer follow-up is needed to investigate postoperative outcomes of patients with GGO lesions. As mentioned above, a larger sample size or longer follow-up may have led to significant survival difference between group C and group D. Fourthly, volumetric measurement is a potential approach in measuring the consolidation as it could provide the profile of total solid components within a nodule. However, volumetric measurement is too early to be applied into clinical practice considering the inconveniency and its dependency on nodule density and segmentation algorithms, etc. Currently, the cutoffs of CTR are still being explored and appropriate grouping could distinguish the prognosis well. Further studies are needed to elaborate the role of volumetric measurement in pulmonary GGOs.

In conclusion, consolidation tumor ratio is an independent prognostic factor for clinical stage IA lung adenocarcinoma manifesting as GGO in CT scan. Radiologic cutoffs of CTR 0.50 and 0.75 were able to subdivide patients with different prognosis. Prospective cohort study is warranted to validate our observations.

## Data Availability Statement

The raw data supporting the conclusions of this article will be made available by the authors, without undue reservation.

## Ethics Statement

This study was approved by the ethics committee on human research of Zhongshan Hospital (approval number: B2019-232R), and written informed consent was obtained from all patients before surgery for the use of surgical samples and clinical information for medical research.

## Author Contributions

JX, JY and JL collected data, conducted the data analysis, interpreted the results, and wrote the main manuscript. CZ, ZL, WJ, SX, and QW designed the research, supervised the data analysis, interpreted the data, and critically revised the article. All authors contributed to the article and approved the submitted version.

## Funding

This study was supported by the National Natural Science Foundation of China (Nos: 81572295). The funding agency played no part in study design, data analysis, interpretation of data, or manuscript preparation.

## Conflict of Interest

The authors declare that the research was conducted in the absence of any commercial or financial relationships that could be construed as a potential conflict of interest.
